# Inhibitory Effects of Ethyl Acetate Extract of *Andrographis paniculata* on NF-**κ**B Trans-Activation Activity and LPS-Induced Acute Inflammation in Mice

**DOI:** 10.1093/ecam/nep120

**Published:** 2011-02-14

**Authors:** Wen-Wan Chao, Yueh-Hsiung Kuo, Shie-Liang Hsieh, Bi-Fong Lin

**Affiliations:** ^1^Department of Biochemical Science and Technology, Institute of Microbiology and Biochemistry, College of Life Science, National Taiwan University, Taiwan; ^2^Department of Chemistry, National Taiwan University, Taipei, Taiwan; ^3^Tsuzuki Institute for Traditional Medicine, College of Pharmacy, China Medical University, Taichung, Taiwan; ^4^Department and Institute of Microbiology and Immunology, National Yang-Ming University, Taiwan

## Abstract

This study was to investigate anti-inflammatory effect of *Andrographis paniculata* (Burm. f.) Nees (Acanthaceae) (AP). The effects of ethyl acetate (EtOAc) extract from AP on the level of inflammatory mediators were examined first using nuclear factor kappa B (NF-**κ**B) driven luciferase assay. The results showed that AP significantly inhibited NF-**κ**B luciferase activity and tumor necrosis factor **α** (TNF-**α**), interleukin 6 (IL-6), macrophage inflammatory protein-2 (MIP-2) and nitric oxide (NO) secretions from lipopolysaccharide (LPS)/interferon-**γ** stimulated Raw264.7 cells. To further evaluate the anti-inflammatory effects of AP *in vivo*, BALB/c mice were tube-fed with 0.78 (AP1), 1.56 (AP2), 3.12 (AP3) and 6.25 (AP4) mg kg^−1^ body weight (BW)/day in soybean oil, while the control and PDTC (pyrrolidine dithiocarbamate, an anti-inflammatory agent) groups were tube-fed with soybean oil only. After 1 week of tube-feeding, the PDTC group was injected with 50 mg kg^−1^ BW PDTC and 1 h later, all of the mice were injected with 15 mg kg^−1^ BW LPS. The results showed that the AP1, AP2, AP3 and PDTC groups, but not AP4, had significantly higher survival rate than the control group. Thus, the control, AP1, AP2, AP3 and PDTC groups were repeated for *in vivo* parameters. The results showed that the AP and PDTC groups had significantly lower TNF-**α**, IL-12p40, MIP-2 or NO in serum or peritoneal macrophages and infiltration of inflammatory cells into the lung of mice. The AP1 group also had significantly lower MIP-2 mRNA expression in brain. This study suggests that AP can inhibit the production of inflammatory mediators and alleviate acute hazards at its optimal dosages.

## 1. Introduction

*Andrographis paniculata* (Burm. F.) Nees (Acanthaceae) (AP), also known as *Chuan-Chin-Lian* in Chinese, is a traditional medicine herb used for the treatment of infection, inflammation, cold, fever, diarrhea and snake-bite as an antidote [1]. Its active constituents such as andrographolide, neoandrographolide and 14-deoxy-11,12-didehydroandrographolide have been studied for anti-inflammatory, anti-cancer and cardiovascular effects [2–4]. Andrographolide, recognized as the most medicinally active phytochemical in AP, has been reported to inhibit lipopolysaccharide (LPS)-induced nitric oxide (NO) production through suppression of inducible nitric oxide synthase (iNOS), and nuclear factor kappa B (NF-*κ*B) activation by blocking the binding of NF-*κ*B oligonucleotide to nuclear proteins [5, 6].

Our previous study also indicated that NF-*κ*B dependent luciferase reporter assay may serve as pre-screen tool to identify anti-inflammatory Chinese medicinal herbs extracted by hexane, ethyl acetate (EtOAc) or H_2_O [7]. Among these different fractions, the EtOAc extract from AP, one of those 22 selected traditional Chinese medicine herbs screened out by suppression of NF-*κ*B luciferase activity, also decreased NO and PGE_2_ production in LPS/interferon-*γ* (IFN-*γ* stimulated macrophage cell line [8]), suggesting that AP is worth for further *in vivo* evidence-based research on anti-inflammatory effect.

*In vivo* inflammatory progresses are mediated by the involvement of pro-inflammatory mediators, including tumor necrosis factor *α* (TNF-*α*), interleukin 6 (IL-6), IL-12, IFN-*γ*, NO and macrophage inflammatory protein-2 (MIP-2). The proper production of these cytokines helps the innate immune response, but an overproduction results in acute phase endotoxemia and causes tissue injury, septic shock and even death [9]. Sepsis is generally considered a systemic inflammatory disorder and a serious clinical problem with high mortality [10]. Injection of LPS in murine model induces acute inflammation, with increased mRNA levels of pro-inflammatory mediators such as TNF-*α*, IL-6, IL-1*β* and MIP-2 in the brain and lung [11, 12].

Hence, the present study was to evaluate the anti-inflammatory effect of the EtOAc extract from AP on *in vitro* NF-*κ*B-dependent reporter activity and *in vivo* LPS-induced acute inflammatory murine model.

## 2. Methods

### 2.1. Extraction and Semi-Purification of AP

AP was purchased from a licensed Chinese herbal drug store in Taipei City, and was authenticated by Wei-Chu Li, PhD (Sheng Chang Pharmaceutical, Co., Ltd, Taiwan) [8]. In total, 10 g of AP was extracted with 300 ml of 95% ethanol at 50°C for 3 h twice. The total crude extract was evaporated under vacuum to yield a residue, and then the residue was suspended in 90% ethanol and successively partitioned with hexane (three times) and ethyl acetate (three times) to obtain hexane, EtOAc and water fractions, respectively.

### 2.2. Transient Transfection and Luciferase Activity Assay

To investigate the activity of NF-*κ*B trans-activation, we used the p3x-*κ*B luciferase reporter assay system. A reporter plasmid, 3x-*κ*B-tk-luc, has three repeats of the NF-*κ*B site upstream of a minimal thymidine kinase promoter and a luciferase reporter gene in pGL_2_ vector (Promega Corp., Madison, WI, USA) [13]. The murine macrophage cell line Raw264.7 were grown in Dulbecco's Modified Eagle Medium (DMEM) supplemented with 10% fetal bovine serum (GIBCO, Grand Island, NY, USA) seeded on 24-well plates (Nunc, Roskilde, Denmark) at a concentration of 5 × 10^4^ cells/well, then incubated for overnight. The cells were cotransfected with 0.3 *μ*g of the NF-*κ*B-promoted luciferase reporter gene plasmid pNF-*κ*B-Luc and 0.1 *μ*g of Renilla luciferase reporter plasmid pRL-tk (Promega) for 48 h using the ExGen 500 *in vitro* transfection reagent (Fermentas, Hanover, MD, USA). After transfection, cells were pre-incubated with AP EtOAc extract or NF-*κ*B-DNA binding inhibitor Helenalin (10 *μ*M, Calbiochem-Novabiochem Corp., San Diego, USA) for 1 h and then stimulated with LPS (100 ng ml^−1^, Sigma, St Louis, MO, USA) plus IFN-*γ* (1000 U ml^−1^, Sigma) for 8 h. Supernatant were collected for cytokines assay. Luciferase expression was then analyzed using the Dual-Glo luciferase reporter assay system (Promega) as previously reported [7]. The cytotoxicity of the AP EtOAc extract (2.5–20 *μ*g ml^−1^), was determined by 3-(4,5-dimethylthiazol-2-yl]-2,5-diphenylterazolium bromide, MTT, Sigma) assay for cell viability [8].

### 2.3. AP Supplement Prior to LPS-Induced Inflammation in Mice

Fifty-four-week-old female BALB/c mice were purchased from the Animal Center of the College of Medicine at National Taiwan University (Taipei, Taiwan). At age of 8 weeks, mice (20 ± 2 g) were divided into six groups: the LPS group (*n* = 8, as a control) and the PDTC group (*n* = 8, as a positive control) were tube-fed with 100 *μ*l soybean oil/day, while the AP groups (*n* = 8/group) were tube-fed with different doses of AP EtOAc extract (AP1, 0.78 mg kg^−1^ body weight (BW); AP2, 1.56 mg kg^−1^ BW; AP3, 3.12 mg kg^−1^ BW and AP4, 6.25 mg kg^−1^ BW) in 100 *μ*l soybean oil/day. The mice had free access to AIN-76 diet and water and additionally tube-fed with either soybean oil or AP EtOAc extract daily. After 1 week of tube feeding, all of the mice were injected i.p. with 15 mg kg^−1^ BW LPS to induce acute inflammation. Mice in the PDTC group were injected i.p. with 50 mg kg^−1^ BW PDTC (Sigma) 1 h before LPS challenge. This PDTC dose were referred to previous studies showing anti-inflammatory effects in LPS-challenged model [14, 15]. The life spans of these mice that continued on AIN-76 diet *ad lib* were recorded.

In the second experiment, the LPS, PDTC, AP1, AP2, AP3 were repeated to further investigate the inflammatory cytokines production (*n* = 8/group). A negative control, the PBS group in this experiment, was injected with PBS instead of LPS. Sera were collected by retro-orbital bleeding at 2 and 6 h after LPS challenge for cytokine assay. Animal care and handling conformed to accepted guidelines [16], and approved by the Institutional Animal Care and Use Committee NTU (IACUC approval No. 95-072).

### 2.4. Collection and Culture of Peritoneal Macrophages

To harvest the female BALB/c mice peritoneal macrophages, all groups of mice in the second experiment were sacrificed by excessive exposure to anesthetic ether at 6 h after LPS challenge. Peritoneal fluid was collected by i.p. injection of 9 ml of cold Hank's balanced salt solution (HBSS; Life Technologies, Paisley, UK). The peritoneal macrophages were centrifuged and were grown in RPMI-1640 medium (Hyclone, Logan, UT, USA) supplemented with 2% TCM (mouse serum replacement, Celox Corp., Hopkins, MN, USA) and antibiotic-antimycotic (Atlanta Biologicals, Norcross, GA, USA). Cells were inoculated in 48-well plates (Nunk) at a concentration of 3 × 10^6^ cells/well for 48 h and the culture supernatant was collected and frozen at −70°C for the cytokines assay.

### 2.5. Lung Histology

To evaluate the effects of AP on LPS-challenged lung inflammation, the lung of mice were immediately removed and inflated fixed with 10% paraformaldehyde. Paraffin embedded lungs were sectioned at 3 *μ*m and stained with hematoxylin and eosin for morphologic analysis.

### 2.6. Cytokine Measurements

The productions of TNF-*α*, IL-6, IL-12p40, MIP-2 and NO in Raw264.7 macrophage cell supernatants and serum and peritoneal macrophages of LPS-challenged mice were assayed by commercial ELISA kits. IL-6 ELISA kit (PharMingen, San Diego, CA, USA) and TNF-*α*, IL-12p40, MIP-2, total NO kits (R&D Systems, Inc., Minneapolis, MN, USA) were used and the cytokine concentration was assayed according to the manufacturer's cytokine ELISA protocol. Detection sensitivity for NO is 1 *μ*M.

### 2.7. Reverse Transcription Polymerase Chain Reaction for Cytokine Gene Expression

Detection of MIP-2 mRNA expression was performed by reverse transcription polymerase chain reaction (RT-PCR). Hepatic and brain TNF-*α* and iNOS mRNA expression were also measured but not significantly different among groups. Liver and brain tissue samples were collected from animals sacrificed at 6 h after LPS challenge. Total RNA was isolated by the TRIzol method (Life Technologies, GIBCO-BRL, Gaithersburg, MD, USA) according to the manufacturer's instructions. PCR primers for MIP-2 and *β*-actin contained the following sequences: MIP-2 sense (5′-TGG GTG GGA TGT AGC TAG TTC C-3′) and anti-sense (5′-AGT TTG CCT TGA CCC TGA AGC C-3′) and *β*-actin sense (5′-ATG GAG AAA ATC TGG CAC CA-3′) and anti-sense (5′-AGT CCA TCA CGA TGC CAG TG-3′). Equal amounts of RNA (1 *μ*g) were reverse-transcribed into cDNA using oligo (dT) 15 primers, 10 *μ*l reaction mixture which containing 1 *μ*l dNTP mixture (10 mM), 0.25 *μ*l RNase inhibitor (Promega) using M-MLV reverse transcriptase (Promega), then amplified using rTaq DNA polymerase (Takara, Japan) in the presence of a sense/antisense primer pair specific for the coding region of the mouse cDNA sequence. The PCR conditions were as follows: 94°C, 1 min; 55°C, 1 min; 72°C, 1 min for 30 cycles. The amplified PCR products were subjected to electrophoresis at 100 V through 1.5% agarose gel for about 30 min. Photographs and scans were analyzed with the BioSpectrum imaging system (UVP, Cambridge, UK).

### 2.8. Statistical Analysis

The data are expressed as the mean ± standard deviation (SD) or mean ± standard error of mean (SEM). The significant difference compared with the control group was statistically by the Student's *t*-test using the SAS software program (SAS/STATA version 8.0; SAS Institute, Cary, NC, USA). Statistical comparison between different survival curves was analyzed by Cox's proportional hazards regression test (STATA version 9.0; Stata Corp., TX, USA). The correlation was analyzed by simple correlation of the SAS program. Statistical significance is expressed as *P* < .05.

## 3. Results

### 3.1. AP EtOAc Extract Suppresses NF-*κ*B Transcriptional Activity and Pro-Inflammatory Mediators Production in LPS/IFN-*γ* Activated Macrophages

**Figure 1:**
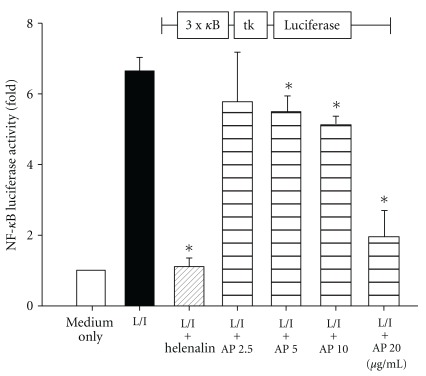
Effect of the EtOAc extract of AP on NF-*κ*B dependent luciferase reporter activity in Raw264.7 cells activated by LPS/IFN-*γ* (L/I). Raw264.7 macrophages transient transfected with a NF-*κ*B reporter plasmid were pretreated with 2.5–20 *μ*g ml^−1^ of AP EtOAc extract or helenalin (NF-*κ*B inhibitor, 10 *μ*M) for 1 h and then stimulated with LPS 100 ng ml^−1^/IFN-*γ* 1000 U ml^−1^ for 8 h. The activity of NF-*κ*B was estimated by the Dual-Glo Luciferase reporter assay. The data are expressed as the mean ± SD from three independent experiments. (Asterisk indicates the significant as compared with the control group (L/I), *P* < .05, the Student's *t*-test).

Activation of Raw264.7 macrophages with LPS + IFN-*γ* resulted in a significant increase in NF-*κ*B trans-activation activity. No cytotoxicity of AP EtOAc extract up to 20 *μ*g ml^−1^ was confirmed by cell viability (data not shown). To test whether AP EtOAc extract pretreatment may involve NF-*κ*B activation, we performed a reporter gene assay with 3x-*κ*B-tk-luciferase transfected into Raw264.7 macrophages (Figure 1). Addition of NF-*κ*B binding inhibitor, Helenalin, thoroughly suppressed reporter activity. AP EtOAc extract pretreatment significantly decreased LPS/IFN-*γ* activated NF-*κ*B luciferase activity in a dose dependent manner (*P* < .05).

The levels of TNF-*α*, IL-6, MIP-2, IL-12p40 and NO in LPS/IFN-*γ*-stimulated Raw264.7 macrophages cell supernatants were also measured (Table 1). Level of IL-12p40 was undetectable in our study (data not shown). The pretreatment with AP EtOAc extract significantly decreased TNF-*α*, IL-6, MIP-2 and NO productions in LPS/IFN-*γ*-stimulated Raw264.7 macrophages compared with LPS/IFN-*γ* treatment only. There was a significant correlation between NF-*κ*B luciferase activity and MIP-2 level (*r* = 0.85, *P* < .001).

### 3.2. Low Doses of AP EtOAc Extract Significantly Improved Survival of LPS-Challenged Mice

**Figure 2:**
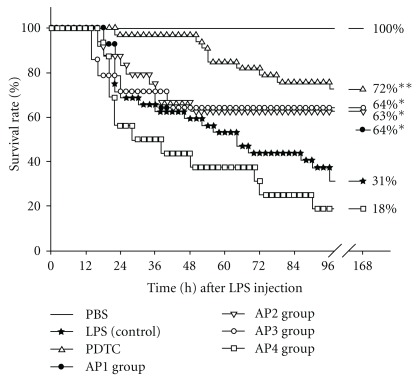
Effects of EtOAc extract of AP on survival rate of mice treated with LPS endotoxin. The mice were divided into the following groups: the PBS, LPS and PDTC groups and the AP 1, 2, 3, 4 groups (tube-feeding 0.78, 1.56, 3.12 and 6.25 mg kg^−1^ BW AP EtOAc extract in 100 *μ*l soybean oil/day, resp.). After 1-week tube-feeding of AP EtOAc extract, all mice were i.p. injected with 15 mg kg^−1^ BW LPS to induce acute inflammation. Mice in the PDTC group were i.p. injected with 50 mg kg^−1^ BW PDTC 1 h before LPS administration. Statistical comparison between different survival curves was analyzed by Cox's proportional hazards regression test. Statistical analysis was performed using the Student's *t*-test for multiple comparisons (Asterisk indicates the significant as compared with LPS alone, *P* < .05, *P* < .01).

To investigate whether suppression of *in vitro* pro-inflammatory cytokines production may benefit survival of LPS-challenged mice, the life spans were recorded (Figure 2). The survival rate in the positive control PDTC group (72%) was significantly higher than that in the control LPS group (31%, *P* < .01). The similar survival rate in the AP1, AP2 and AP3 groups (64, 63 and 64%, resp.) were also significantly higher than that of the LPS group (31%) according to the Cox proportion hazards regression test. Overall, the AP4 group had low survival rate (18%).

### 3.3. Suppression of TNF-*α*, IL-6, IL-12p40, MIP-2 and NO Levels in Serum and Peritoneal Exudates Macrophages from LPS-Challenged Mice Pretreated with AP EtOAc Extract

To investigate the *in vivo* anti-inflammatory effect of AP EtOAc extract, levels of pro-inflammatory cytokines in serum were determined at 2 and 6 h after LPS challenge in mice (Table 2). Our data showed that mice injected with LPS had marked increase of serum TNF-*α*, IL-6, IL-12p40, MIP-2 and NO, while mice in the AP and PDTC groups showed significantly lower serum TNF-*α* levels at 2 h after LPS challenge, compared with the LPS group. The PDTC and AP2 groups also had significantly lower TNF-*α* levels in serum and peritoneal macrophages at 6 h after LPS challenge. IL-6 levels in serum and peritoneal macrophages did not significantly decrease in AP EtOAc extract groups as compared with LPS group at 2 and 6 h after LPS injection, which may be due to individual variations.

With regard to IL-12p40, a subunit of the heterodimeric cytokine, LPS challenge induced an increase in its level in serum, which was decreased by PDTC and AP especially significantly after 2 h challenge. AP2 group also had significantly lower IL-12p40 production from peritoneal macrophages compared to the LPS group.

Serum MIP-2 levels rose at 2 h and dropped at 6 h after LPS challenge and were suppressed by PDTC and AP, significantly in the PDTC and AP1 groups at 2 h after challenge (Table 2). The PDTC and AP2 groups also had significantly lower MIP-2 production from peritoneal macrophages.

Serum levels of NO was detectable only at 6 h after LPS challenge, and was significantly lower in the AP1 and AP2 groups compared with the LPS group. The NO production by peritoneal macrophages was significantly suppressed by PDTC, and by AP supplementation in a dose-dependent manner.

### 3.4. Inhibition of MIP-2 mRNA Expression in Brain by AP EtOAc Extract

**Figure 3:**
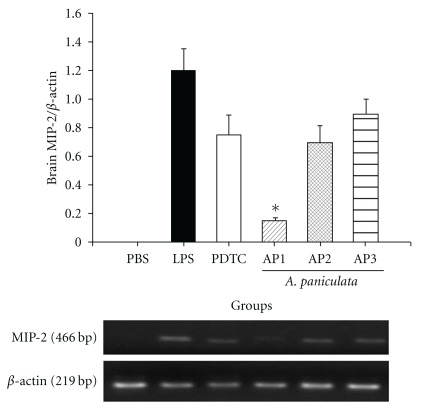
Effects of EtOAc extract on endotoxin induced MIP-2 mRNA expression in brain of BALB/c mice. The mice were divided into the following groups: the PBS, LPS and PDTC groups and the AP 1, 2, 3 groups (tube-feeding 0.78, 1.56 and 3.12 mg kg^−1^ BW AP EtOAc extract in 100 *μ*l soybean oil/day, resp.). After 1-week tube-feeding of AP EtOAc extract, all mice were i.p. injected with 15 mg kg^−1^ BW LPS to induce acute inflammation. Mice in the PDTC group were i.p. injected with 50 mg kg^−1^ PDTC 1 h before LPS administration. Results of MIP-2/*β*-actin mRNA ratio are expressed as mean ± SEM (*n* = 8). Statistical analysis was performed using Student's *t*-test for multiple comparisons (Asterisk indicates the significant as compared with LPS alone, *P* < .05).

To investigate whether AP affect mRNA expression, the mRNA expression of MIP-2, TNF-*α* and iNOS in liver and brain at 6 h after LPS challenge was measured. Though TNF-*α*, iNOS and hepatic MIP-2, mRNA expression were not found significantly affected by PDTC or AP (data not shown), MIP-2 mRNA in brain was suppressed by PDTC and AP, especially significantly in the AP1 group compared with the LPS group (Figure 3).

### 3.5. AP EtOAc Extract on Inflammatory Cell Infiltration

**Figure 4:**
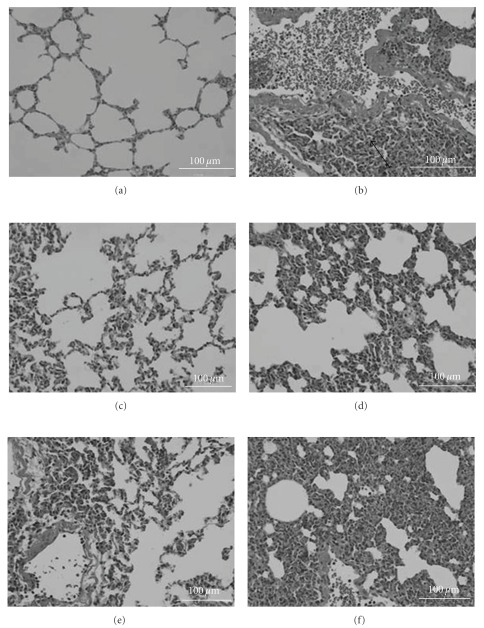
Morphological changes in mouse lung sections on LPS induction of sepsis with/without treatment with EtOAc extract of AP. Histopathologic analysis was performed on hematoxylin and eosin (H. E.) stained sections of lung at 6 h after LPS administration. (a) PBS group was received an i.p. injection of PBS buffer; (b) LPS group, received an i.p. injection of 15 mg kg^−1^ BW LPS severe lung edema and inflammatory cells infiltration (arrow) were observed in this group; (c) PDTC + LPS group, received an i.p. injection of 50 mg kg^−1^ BW PDTC 1 h before LPS administration; (d) AP 1 group, was given an oral dose of 0.78 mg kg^−1^ BW AP; (e) AP 2 group, was given an oral dose of 1.56 mg kg^−1^ BW AP; (f) AP 3 group, was given an oral dose of 3.12 mg kg^−1^ BW AP. (magnification 400x).

As illustrated in Figure 4, the lung of mice injected with LPS showed marked inflammation characterized by infiltration of inflammatory cells into the alveolar space, a thickening of the alveolocapillary membrane, and the presence of alveolar hemorrhage (Figure 4(b)) when compared with those of mice injected with PBS (Figure 4(a)). In contrast, histological damage was less pronounced in PDTC or AP-treated mice (Figures 4(c)–4(e)).

## 4. Discussion

To investigate the potential of bioactive chemicals contained in natural health products as effective drug therapy, methods involving gene expression analysis, cell membrane chromatography or trans-activated reporter gene assay, have been developed and proposed as rapid throughput screening systems [8, 17–19]. Our previous study on pre-screening of 22 commonly used Chinese herbs by NF-*κ*B-dependent activity implies that AP EtOAc extract exerts an anti-inflammatory effect [7]. In this study, AP EtOAc extract was confirmed not only to inhibit NF-*κ*B activation dose-dependently (Figure 1), but also TNF-*α*, IL-6, MIP-2 and NO production in LPS/IFN-*γ* stimulated Raw264.7 macrophages (Table 1). NF-*κ*B activation is a central event leading to the activation of complex cytokine and inflammatory mediator network in septic shock and inflammation. The activation of NF-*κ*B results in the expression of genes encoding inflammation mediators such as TNF-*α*, IL-6, MIP-2 and NO [20], which were suppressed by PDTC and AP in this study.

TNF-*α* is the cytokine as the target for development of new medications development for disease involving inflammation, such as rheumatoid arthritis. Inhibition of elevated TNF-*α* induced by LPS has been a common practice for evaluation of anti-inflammatory effect of drug candidates [21, 22]. Our results showed lower production of TNF-*α* not only in LPS-stimulated Raw264.7 cells pretreated by AP but also in serum and from activated peritoneal macrophages of LPS-challenged mice supplemented with AP, confirming TNF-*α* as a target in anti-inflammatory strategy. IL-6 is also an important mediator of inflammation. The secretion of IL-6 by activated Raw264.7 cells was the most significantly inhibited among the cytokines measured in this study. However, no significance effect was observed in LPS-challenged mice. It may due to the efficacy of dosages applied *in vivo* or different experimental animal model of sepsis used [23].

Another cytokine, IL-12p40, was recently shown that its absence alleviated the chronic phase of arthritis and reduced iNOS levels, suggesting that IL-12p40 plays a critical role in late phase of inflammation [24]. In this study, activated Raw264.7 cells produced minimal IL-12p40, while LPS challenge to mice induced its production in serum and peritoneal cells and was suppressed by PDTC and AP. NO is an important mediator in both acute and chronic inflammation. The inflammatory responses are largely controlled through regulation of NF-*κ*B [6]. The significant decrease of NO production by both Raw264.7 cells and LPS-challenged mice confirmed our previous report of the association of NO production and NF-*κ*B-dependent reporter activity [8].

Although the suppression did not necessarily reach statistically significance in all the parameters measured in this study, the survival rate in murine endotoxin shock, a director indicator for anti-inflammation, suggested a link of test samples *in vitro* potency with its *in vivo* activities. Further analysis of the correlation between life span and serum cytokine levels indicated that life span negatively correlated with serum TNF-*α* and MIP-2 levels (*r* = −0.60, *P* < .001 for TNF-*α*; *r* = −0.56, *P* = .03 for MIP-2) at 2 h after LPS-challenge, and serum NO levels (*r* = −0.58, *P* = .02) at 6 h after LPS-challenge. This implies that suppression of TNF-*α* and MIP-2 in the early stage of acute-inflammation may prolong the life of mice suffering from endotoxic shock, and inhibition of NO production in the late stage still benefit animal survival.

It has been shown that MIP-2 mRNA expression can be induced tissue-specifically in lung, brain, kidney and heart in endotoxemia mice [25]. Brain plays a key role in modulating the immune response, and inappropriate bilateral interplay between the central nervous and immune systems may have detrimental consequences during infection and inflammatory processes. Thus, brain MIP-2 mRNA were also detected in our study (Figure 3). MIP-2 in mouse hypothalamus induces neutrophil infiltration and permeabilizes the blood-brain barrier thus permitting the entry of leukocytes across the parenchyma [26], suggesting that MIP-2 play an important role in neutrophil accumulation in brain tissues [27].

Our analysis of relationship between NF-*κ*B luciferase activity and these inflammatory mediators obtained a significant correlation between NF-*κ*B luciferase activity and MIP-2 level in activated Raw264.7 cells. MIP-2, a critical chemokine for neutrophils recruitment, is secreted by macrophage and epithelial cells in response to inflammatory stimuli, such as LPS [28]. Its expression induced by LPS is dependent on NF-*κ*B activation [29], and important for neutrophil accumulation in inflammatory response in lung [30]. Our *in vivo* study also demonstrated that LPS-challenged mice in the AP1, AP2 and PDTC groups that had lower MIP-2 in serum or produced from peritoneal macrophage, mRNA expression in brain, and also less inflammatory cell infiltration in lung (Figure 4). These results are in accordance with the higher survival rate in these groups, suggesting that suppression of MIP-2 production is an important indicator for alleviation of inflammatory process.

LPS released during bacterial infections induce the expression of pro-inflammatory cytokines. Systemic inflammation leads to inflammatory responses in the brain. The secretion of cytokines and inflammatory mediators induce sickness behavior characterized by fever, lethargy, hypophagia, anxiety and depression, and so forth, [31]. In traditional Chinese medicine, AP is used to get rid of the body heat, for example, fevers and acute infections and to dispel toxins from the body. AP was shown to inhibit the pro-inflammatory chemokine RANTES secretion by human bronchial epithelial cells infected with influenza A virus H1N1 [32]. Our results demonstrate that AP can also inhibit MIP-2 mRNA expression. Mechanism of action of MIP-2 on inflammation in brain is less documented compared with those of cytokines and chemokines, such as TNF-*α*, IL-1*β*, MCP-1 and MIP-1, though they all increase in both the periphery and in the brain when LPS is administered peripherally [33, 34]. The identification of bioactive compounds in AP EtOAc extract and its significance of MIP-2 suppression on inflammation are worthy of investigation. The isolation and identification of anti-inflammatory effect of these compounds are undergoing in our laboratory. By bioactivity-guided chromatographic fractionation, the ratio of the bioactive compounds is 23% of the AP EtOAc extract, with major compounds such as andrographolide (12%), 14-deoxy-11,12-didehydroandrographolide (6.8%) and ergosterol peroxide (3.2%) that exerting significant inhibition of NF-*κ*B trans-activation.

However, the AP 4 group with higher dose of AP EtOAc extract showed lower survival rate (Figure 2). Although further studies are required to clarify the reasons, it is known that traditional Chinese medicine have their optimal effective dosage and overdose may cause side-effect or toxicity. Studies of acute toxicity in mice reported a lethal dose (LD50) of some components of AP, such as diterpene lactones, deoxyandrographolide, neoandrographolide, dehydroandrographolide and andrographolide [35], suggesting that traditional herb medicine has its optimal and effective dosages [36].

**Figure 5:**
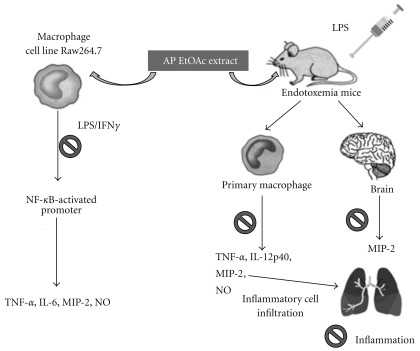
*In vitro* and *in vivo* biological actions of ethyl acetate extract of AP as an anti-inflammatory traditional medicine herb. Prohibition sign indicate the inhibitory effect of ethyl acetate extract of AP.

In conclusion, pretreatment of AP EtOAc extract that inhibit NF-*κ*B trans-activation activity and pro-inflammatory cytokines *in vitro*, protects mice against sub-lethal endotoxic shock. Pro-inflammatory cytokines such as TNF-*α*, IL-12p40, MIP-2 and NO in serum and activated peritoneal macrophage are significantly inhibited, and thus the survival rates of LPS-challenged mice are improved by AP EtOAc extract at the optimal dosages (Figure 5).

## Figures and Tables

**Table 1:** Effects of AP EtOAc extract on pro-inflammatory cytokines production from LPS/IFN-*γ* stimulated Raw264.7 macrophage cells.

Raw264.7 macrophage cells				
Treatment	TNF-*α* (pg ml^−1^)	IL-6 (pg ml^−1^)	MIP-2 (ng ml^−1^)	NO (*μ*M)
Medium only	n.d.	n.d.	n.d.	1.24 ± 0.25
LPS/IFN-*γ* (L/I)	717 ± 218	410 ± 95	1.48 ± 0.48	6.95 ± 1.21
L/I + Helenalin	n.d.	n.d.	n.d.	2.62 ± 0.62*
L/I + AP 2.5	667 ± 208	282 ± 98*	1.49 ± 0.72	3.29 ± 0.88*
L/I + AP 5	509 ± 185	275 ± 65*	1.39 ± 0.53	3.08 ± 0.80*
L/I + AP 10	388 ± 115*	100 ± 40*	1.16 ± 0.48*	3.03 ± 0.73*
L/I + AP 20	348 ± 62*	50 ± 10*	0.35 ± 0.05*	2.87 ± 0.73*

The data are expressed as the mean ± SD from three independent experiments. The significant difference compared with the control group was statistically by the Student's *t*-test (significant as compared with L/I alone, *P* < .05). Raw264.7 macrophages transient transfection with a NF-*κ*B reporter plasmid were pretreated with 2.5–20 *μ*g ml^−1^ of AP EtOAc extract or helenalin (NF-*κ*B inhibitor, 10 *μ*M) for 1 h and then stimulated with L/I for 8 h. Supernatant were collected and analyses. n.d.: not detectable.

**Table 2:** Effects of AP EtOAc extract on levels of pro-inflammatory mediators in serum and peritoneal exudates macrophages at 6 h in BALB/c mice treated with LPS.

Groups	TNF-*α* (pg ml^−1^)	IL-6 (ng ml^−1^)	IL-12p40 (ng ml^−1^)	MIP-2 (ng ml^−1^)	NO (*μ*M)
Serum					
2 h					
PBS	n.d.	n.d.	n.d.	n.d.	n.d.
LPS	2325 ± 504	167 ± 14.2	11.5 ± 2.38	140.6 ± 5.3	n.d.
PDTC	737 ± 174*	161 ± 15.8	3.57 ± 0.71*	64.1 ± 3.0*	n.d.
AP1	1175 ± 113*	200 ± 52.0	10.7 ± 1.03	73.8 ± 3.5*	n.d.
AP2	1150 ± 96*	136 ± 11.7	9.84 ± 1.16*	86.8 ± 4.7	n.d.
AP3	2647 ± 1292	145 ± 11.5	2.01 ± 0.35*	110.3 ± 8.0	n.d.
6 h					
PBS	n.d.	n.d.	n.d.	n.d.	n.d.
LPS	660 ± 62	295 ± 38.3	23.7 ± 2.66	34.4 ± 3.1	234 ± 34.8
PDTC	403 ± 38*	148 ± 30.6	11.7 ± 0.80*	24.0 ± 2.6	192 ± 33.8
AP1	483 ± 31	110 ± 30.6	24.1 ± 2.30	15.1 ± 5.5	85 ± 5.30*
AP2	400 ± 32*	231 ± 35.3	19.3 ± 1.97	28.7 ± 2.9	101 ± 17.5*
AP3	434 ± 55	333 ± 51.5	18.7 ± 2.08	27.7 ± 5.6	310 ± 34.4
Peritoneal macrophages					
6 h					
PBS	n.d.	n.d.	n.d.	n.d.	n.d.
LPS	158 ± 13	37.9 ± 7.3	40.4 ± 6.7	14.4 ± 1.0	53.3 ± 1.7
PDTC	89 ± 6.1*	30.6 ± 6.9	31.2 ± 5.1	6.7 ± 0.8*	22.7 ± 4.7*
AP1	102 ± 12	53.5 ± 7.5	40.0 ± 4.0	13.6 ± 1.3	39.7 ± 4.0*
AP2	89 ± 6.1*	41.4 ± 8.9	24.8 ± 2.7*	6.3 ± 0.5*	33.4 ± 4.6*
AP3	238 ± 53	17.3 ± 3.0	30.2 ± 4.5	7.6 ± 1.0	24.5 ± 4.3*

The mice were divided into the following treatment groups: LPS group; PDTC group; AP1 group, a dose of 0.78 mg kg^−1^ BW AP; AP2 group, a dose of 1.56 mg kg^−1^ BW AP; AP3 group, a dose of 3.12 mg kg^−1^ BW AP. Treatments were administrated orally 7 days before LPS challenge (15 mg kg^−1^, i.p.). Data are expressed as mean ± SD of eight mice. Statistical analysis was performed using the Student's *t*-test (significant as compared with LPS alone, * *P* < .05). n.d.: not detectable.
